# A survey of patient perspectives on the research use of health information and biospecimens

**DOI:** 10.1186/s12910-016-0130-4

**Published:** 2016-08-15

**Authors:** Stacey A. Page, Kiran Pohar Manhas, Daniel A. Muruve

**Affiliations:** 1 Department of Community Health Sciences, Cumming School of Medicine, University of Calgary, TRW Building, 3rd Floor, 3280 Hospital Drive NW, Calgary, AB T2N 4Z6 Canada; 2 Conjoint Health Research Ethics Board, Research Services, University of Calgary, MacKimmie Library Tower, 3rd Floor, 2500 University Drive NW, Calgary, AB T2N 1N4 Canada; 3 Alberta Centre for Child, Family & Community Research, Child Development Centre, 2888 Shaganappi Trail NW, Calgary, AB T3B-6A8 Canada; 4 Department of Medicine, Division of Nephrology and Hypertension, Snyder Institute for Chronic Diseases, University of Calgary, 3280 Hospital Dr. NW, Calgary, AB T2N 4Z6 Canada

**Keywords:** Health information, Biospecimens, Informed consent, Secondary use, Research ethics, Privacy

## Abstract

**Background:**

Personal health information and biospecimens are valuable research resources essential for the advancement of medicine and protected by national standards and provincial statutes. Research ethics and privacy standards attempt to balance individual interests with societal interests. However these standards may not reflect public opinion or preferences. The purpose of this study was to assess the opinions and preferences of patients with kidney disease about the use of their health information and biospecimens for medical research.

**Methods:**

A 45-item survey was distributed to a convenience sample of patients at an outpatient clinic in a large urban centre. The survey briefly addressed sociodemographic and illness characteristics. Opinions were sought on the research use of health information and biospecimens including consent preferences.

**Results:**

Two hundred eleven of 400 distributed surveys were completed (response rate 52.8 %). Respondents were generally supportive of medical research and trusting of researchers. Many respondents supported the use of their information and biospecimens for health research and also preferred consent be sought for use of health information and biospecimens. Some supported the use of their information and biospecimens for research without consent. There were significant differences in the opinions people offered regarding the research use of biospecimens compared to health information. Some respondent perspectives about consent were at odds with current regulatory and legal standards.

**Conclusions:**

Clinical health data and biospecimens are valuable research resources, critical to the advancement of medicine. Use of these data for research requires balancing respect for individual autonomy, privacy and the societal interest in the greater good. Incongruence between some respondent perspectives and the regulatory standards suggest both a need for public education and review of legislation to increase understanding and ensure the public’s trust is maintained.

**Electronic supplementary material:**

The online version of this article (doi:10.1186/s12910-016-0130-4) contains supplementary material, which is available to authorized users.

## Background

Research using personal health information and biospecimens is central to the advancement of medicine. Electronic health records and clinical biobanks are rich data sources that can be used for secondary, research purposes. In Canada, academic researchers affiliated with institutions receiving federal funds must comply with the ethics standards articulated in the TriCouncil Policy Statement 2 (TCPS2) [1]. Research using health information must also comply with provincial privacy statues [2].

Disclosure of health information initially collected in a clinical context is regulated by the Health Information Act (HIA) in Alberta and by similar statutes across Canada [2, 3]. The HIA permits disclosure of identifiable health information for research if certain conditions are met, including proposal review by a Research Ethics Board (REB) (s. 49) and consideration of the need for consent (s 50). Where consent is necessary, a number of conditions must be satisfied (s. 34). First, the consent must be provided in writing or electronically. The consent must also contain: an authorization for disclosure, the purpose of the disclosure, the identity of the person to whom it will be disclosed, an acknowledgement that the subject of the information is aware of why the information is needed, the risks and benefits associated with consent, the date on which the consent is effective, the date on which it expires (if any) and lastly, a statement that the consent may be revoked at any time. The HIA does not permit surrogates, such as family members or spouses, to provide consent unless a specific personal (research) directive is in place (s. 104).

In certain circumstances, the REB may determine that consent is not required (s. 50). For consent to be waived, the REB must find that the public interest outweighs the individual privacy interests, the researchers are qualified to carry out the research, privacy and confidentiality safeguards are in place and to obtain consent would be unreasonable, impractical or not feasible.

Characteristics of the research that might preclude consent include extremely large sample sizes, conditions with high mortality rates, or health information from a period in the past where it is likely most of the individuals would be lost to follow up in the present day. Finally, consent is not required where the information released is non-identifying (s 32(1)). Consistent with the law, TCPS2 emphasizes the importance of informed consent, but also identifies circumstances where non-consensual research may be permitted. The two standards differ with respect to the permissibility of surrogate consents and the form the consent must take.

Human biological materials, such as organ/tissue samples, bodily fluids or wastes, are also collected for clinical purposes, but may be accessed for secondary use as research materials. The removal of tissues is regulated in Alberta under the Hospitals Act [4]. Under the Hospitals Act, surgically removed tissue must be submitted to a pathologist and then stored for specified periods. The provincial laboratory retains what it needs for clinical purposes but generally has excess amounts that are repurposed for research use.

In contrast to health information, there are no laws that govern the research use of human biological materials but ethics oversight in the form of REB review is required under the TCPS 2 (2014). Similar to health information, where biospecimens are de-identified, individual consents are not required for their use in research. If the materials are identifiable, researchers must seek an informed consent unless several, specific conditions are met [1].

While these provisions are clear, Canadian legal precedent has found that human tissue removed for medical tests becomes the property of the hospital where the procedure was performed, potentially limiting Canadians’ ability to decide what is done with their biological materials. This is consistent with the situation in the United States, where since the 1990’s patients are considered to have given up their rights to excised tissue. However, proposed new regulations may challenge this [5–7].

Both health information and biological specimens are valuable sources of research data. These resources can be sought on a study-by-study basis directly from individuals, or can be stored in data registries in the case of health information, or biobanks in the case of biological samples. Stewardship of the material then rests with custodians of the registries and biobanks. There has been much written on research use of health information and biospecimens [8–12]. Public opinion with respect to the use of health information and biospecimens is varied, with diversity of opinion reflected in beliefs about consent and related issues [8, 13–17]. Some prefer an opt-out approach where research use is assumed unless people state otherwise. Others prefer a broad, one time consent granting access to all future uses of their information and biosamples, while some prefer to provide consent each time their data are accessed [11, 13, 15, 18].

Considerations influencing individual consent preference include the nature of the data (i.e., health information or biospecimens), who is accessing the information, what the research is about and whether the information is identifiable or not [11, 15, 19, 20]. In addition, the public’s experience with health care and research are likely to influence perspectives as are sociodemographic characteristics including education, gender and age [15–17, 20, 21].

Where consent is required, evidence suggests that those consenting differ systematically from those who decline, biasing research results [9, 22, 23]. Such information is important as research ethics boards and data custodians continue to grapple with how best to balance public rights with individual interests arising from use of these research resources [10, 18]. Understanding public opinion as it reflects the considerations research ethics boards must make, particularly in the context of the Health Information Act and TCPS2 will assist these bodies in discharging their responsibilities. Specific patient groups bring unique perspectives to these considerations.

This survey sought to assess the opinions of patients with chronic kidney disease about the research use of health information and biospecimens.

## Methods

This study received approval from the Conjoint Health Research Ethics Board, University of Calgary (REB15-0095). Survey development was informed by the Health Information Act of Alberta, the TriCouncil Policy Statement 2 (2014) and a literature review. Questions were specific to the research aims and the survey was refined iteratively. Face and content validity were assessed by the research team. The survey was subsequently reviewed by 6 external content experts (legal, ethics, privacy, research administration). Finally, 10 outpatients participated in cognitive interviews about the instrument’s clarity and comprehensibility [24]. Recommendations for revision were discussed and revisions were made based on consensus of the research team.

The final survey (Additional file 1) contained 45 fixed-choice items about patient experiences and opinions regarding health research and use of personal health information and/or biospecimens for research purposes as well as limited socio-demographic and illness information (e.g., gender, age, education, ethnicity, income, illness severity). Prefacing the opinion statements, participants were told to assume their health information/biospecimens would be kept secure with the strongest data and privacy protections. They were also advised that where data were described as de-identified, this meant that researchers would not know from whom the information came. An open-ended text box was provided for participants to comment on consent and research use of their data and/or specimens. Participants were given a one-page background information sheet that described medical research, health information, biospecimens and current regulatory context, including Research Ethics Boards’ roles and responsibilities and HIA provisions (Additional file 2).

Participant recruitment, using convenience sampling, was undertaken for an eight-week period. Adult patients (> = 18 years) presenting for their renal clinic appointment in a large, urban hospital, were asked about their willingness to take part in the survey. Those agreeing were given a package containing the survey, consent, background information and postage-paid return envelope. Participants were invited to return the survey to a box in the clinic waiting area, to any clinic staff member, or via regular mail after leaving the clinic. Return of the survey was taken to be an indication of implied consent as explained in the consent document.

The quantitative results were summarized using frequencies and proportions. The latter were reported as actual percentages, with those not responding to a given item still included in the denominator. To conduct tests for associations, the response options “strongly agree” and “agree” were collapsed into one category and “strongly disagree” and “disagree” were collapsed. Opinions on consent and specific participant characteristics were assessed, using chi-square (SPSS version 21). Opinions about the use of health information compared to use of biospecimens were also compared (chi-squares). The qualitative, textual responses were summarized using descriptive content analysis [25].

## Results

### Response rate

Eight hundred and forty-eight patients were invited to participate, 448 declined. Of the 400 who agreed, 211 returned completed surveys (response rate of 24.9 % overall, response rate of 52.8 % for surveys distributed).

### Sample characteristics

Socio-demographic and health-related characteristics of the participants are summarized in Table 1. Gender was evenly split (46.4 % male, 44.1 % female, 9.5 % preferred not to answer), participants were on average 59 years old and most had some post secondary education (70.2 %). Kidney biopsy status and the length of time participants had been patients at the clinic were used as indicators of illness severity. Using these variables, more than half of the participants could be considered as having chronic illness. Considering past research participation, one quarter of participants indicated they had previously taken part in research. Table 2 provides a description of the research characteristics.

**Table 1:** Participant characteristics

Variable	*N* (% of those responding)
Gender	
male	98 (46.4 %)
female	93 (44.1 %)
preferred not to answer	20 (9.5 %)
Age	
mean	58.9 (SD = 17.3)
median	58
preferred not to answer	27 (12.8 %)
Education	
high school or less	37 (17.5 %)
some post secondary	40 (19.0 %)
technical school/college diploma complete	47 (22.3 %)
post secondary complete	61 (28.9 %)
preferred not to answer/did not answer	26 (12.3 %)
Income	
<= 40 000.00	43 (20.4 %)
$40 000 < $60 000	29 (13.7 %)
$60 000 < $80 000	27 (12.8 %)
$80 000 < $100 000	19 (9.0 %)
>$100 000	33 (15.6 %)
preferred not to answer/did not answer	60 (28.4 %)
Ethnic background	
Canadian	12 (5.0 %)
European Canadian	121 (57.3 %)
African Canadian	5 (2.4 %)
Asian Canadian	13 (6.2 %)
First Nations	3 (1.4 %)
other	27 (12.8 %)
preferred not to answer/did not answer	42 (19.9 %)
Ever had kidney biopsy	
yes	116 (55.0 %)
no	66 (31.3 %)
preferred not to answer	29 (13.7 %)
Affiliated with kidney clinic for	
less than one year	19 (9.0 %)
1–5 years	83 (39.3 %)
6–10 years	28 (13.3 %)
more than 10 years	20 (9.5 %)
first visit	34 (16.1 %)
prefer not to answer/did not answer	27 (12.8 %)

**Table 2:** Characteristics of previous research participation for past participants (*N* = 53)

Research included	*N* (%)
drug or other medical treatment (e.g., counseling, exercise program)	14 (25.9 %)
imaging information (e.g., x-ray, MRI, CT scan)	5 (9.3 %)
tissues samples (e.g., blood, urine, stool, tumor)	12 (22.2 %)
personal health information (e.g., chart, medical records)	25 (47.2 %)
surveys, questionnaires, interviews	20 (37.7 %)
do not remember	10 (18.9 %)

### Health information

Participants were asked to describe the extent to which they agreed with a series of statements about consent, research and health information (Table 3). Almost all participants agreed that promoting medical research was an important goal for the community (97.2 %) and most trusted medical researchers (82.5 %) to use their identifiable information respectfully. Moreover, most (79.6 %) trusted REBs to decide when consent was needed.

**Table 3:** Participant opinions on research, consent and use of health information

Statement	Strongly Disagree %	Disagree %	Neutral %	Agree %	Strongly Agree %
1. Promoting good medical research is an important goal for the community	1.4	0.5	0	31.3	65.9
2. Sometimes medical research cannot be undertaken without using individuals identifiable health information	3.8	5.2	13.7	55.5	19.0
3. I trust Research Ethics Boards to make decisions about when individuals’ consent for use of their identifiable health information for research is necessary	0	2.4	16.6	50.7	28.9
4. Sometimes, getting an individual’s consent to use identifiable health information for research might be so difficult that it would prevent the research from being done	1.9	8.1	18.5	51.7	16.1
5. If consent can only be obtained from some individuals and therefore only some information can be gathered, the research results might not be as useful	0.9	10.4	12.3	53.1	20.4
6. Individuals should always be asked if their de-identified health information can be used for medical research	7.1	11.4	11.4	44.5	22.7
7. Individuals should always be asked if their identifiable health information can be used for medical research	2.8	2.4	12.3	45.0	34.6
8. Consent must be obtained in writing or by electronic means (i.e., by email) for it to be OK	3.8	13.3	17.1	41.7	21.3
9. It is OK to obtain consent verbally (i.e., over the phone, or in fact to face discussion)	6.6	25.1	19.4	37.9	8.5
10. When individuals are unable to give their own consent, it is OK to get consent from a family member	3.8	11.8	15.2	54.5	11.4
11. There are risks from researchers using identifiable health information	4.7	16.6	30.3	39.3	6.6
12. There are benefits from researchers using identifiable health information	0.9	4.7	9.0	53.1	29.4
13. I trust medical researchers to use identifiable health information respectfully	0	0.9	13.7	52.6	29.9

Despite assurances that de-identified data meant their identities would not be known, and despite recognizing that challenges with obtaining consent might compromise research (items 4 and 8) two-thirds of respondents (67.2 %) believed individuals should always be asked if their de-identified information could be used for medical research.

In contrast to the provisions of the HIA, most participants (65.9 %) believed where individuals could not give their own consent, it was permissible to obtain consent from a family member. Also in contrast to the HIA, 46.4 % agreed that a verbal consent was acceptable.

Several variables were examined for their potential influence on consent for use of their identifiable or de-identified information (age, education, ethnicity, experience with research, gender, income, illness severity). Two associations were significant. Participants with less education and those who were younger were more likely to always give researchers consent for use of their identifiable information (*p* = 0.034 and *p* = 0.001 respectively).

Participants were next asked to provide their level of agreement with a series of statements about the use of their health information and biospecimens (Table 4). Although most participants agreed they would always give consent for their identifiable health information (61.1 %) or biospecimens (57.3 %) to be used for any medical research purpose, substantial proportions indicated they would need to know the exact purpose (45.5 % for health information; 29.9 % for biospecimens) before giving consent, or that they would only consent to permitting research on their specific health issues (41.7 % for health information; 37.9 % for biospecimens). Most agreed they should be given the opportunity to change their minds and revoke consent (61.2 % for health information, 61.1 % for biospecimens).

**Table 4:** Participant opinions on research use of health information and biospecimens

Statement	Strongly Disagree %	Disagree %	Neutral %	Agree %	Strongly Agree %
1. I would always give medical researchers consent to use my identifiable health information or biospecimens for any medical research purpose					
health information	4.7	18.0	15.2	43.1	18.0
biospecimen	3.8	14.2	22.7	41.7	15.6
2. I need to know the exact medical research purpose for which my identifiable health information or biospecimen is being used before I would give consent					
health information	9.0	21.8	21.8	33.2	12.3
biospecimen	10.4	34.1	23.2	23.7	6.2
3. It is enough for me to know my identifiable health information or biospecimen is being used for medical research in general					
health information	3.3	15.6	20.4	46.9	12.0
biospecimen	3.8	14.2	17.1	47.4	14.7
4. I would consent to have my identifiable health information or biospecimens held in a research repository, data library or biobank and used as needed by researchers					
health information	1.9	12.8	14.2	50.2	18.0
biospecimen	3.3	9.0	15.2	53.1	17.1
5. I would give consent to use my identifiable health information or biospecimen for research only on my specific health/illness issues					
health information	7.6	28.9	20.4	33.6	8.1
biospecimen	6.6	29.4	23.7	30.8	7.1
6. I need to know the identity of the person to whom my identifiable health information/biospecimen is being given before I would give consent					
health information	11.8	41.2	23.2	18.0	4.3
biospecimen	13.7	44.1	20.9	13.3	5.2
7. I am comfortable with medical researchers using my de-identified health information or biospecimens without my consent					
health information	6.6	16.6	10.9	40.8	22.7
biospecimen	4.3	16.6	16.1	40.3	20.9
8. I believe I should be given the opportunity to change my mind and take back my consent once I have given it					
health information	3.3	11.8	22.3	42.2	19.0
biospecimen	3.8	9.5	22.7	43.1	18.0
9. I would like to know when my identifiable health information or biospecimens are used for medical research					
health information	5.7	16.6	26.5	36.5	13.3
biospecimen	7.6	26.5	20.4	34.6	8.1
10. I would like to know when my de-identified health information or biospecimens are used for medical research					
health information	9.5	28.9	24.6	27.5	7.6
biospecimen	13.3	32.2	19.0	28.0	4.3

Most participants were comfortable with the unconsented use of de-identified health information (63.5 %) or biospecimens (61.2 %). While significant proportions of participants wanted to know when their information or biospecimens were used for research, there was variation that related to the identifiability of the material as well as whether it was health information or biospecimens that were used.

Across items, there were statistically significant differences in the opinions people offered for use of biospecimens compared to health information (Table 5).

**Table 5:** Comparison of participant opinions on research use of health information, biospecimens and consideration of identifiability

Statement	Disagree/Strongly disagree % (*N*)	Agree/Strongly agree % (*N*)
1. I would always give medical researchers consent to use my identifiable health information or biospecimens for any medical research purpose *		
health information	23.1 (48)	62.0 (129)
biospecimen	18.4 (38)	58.7 (121)
2. I need to know the exact medical research purpose for which my identifiable health information or biospecimen is being used before I would give consent *		
health information	31.6 (65)	46.6 (96)
biospecimen	45.9 (94)	30.7 (63)
3. It is enough for me to know my identifiable health information or biospecimen is being used for medical research in general *		
health information	19.4 (40)	60.2 (124)
biospecimen	18.6 (38)	64.2 (131)
4. I would consent to have my identifiable health information or biospecimens held in a research repository, data library or biobank and used as needed by researchers *		
health information	15.1 (31)	70.5 (144)
biospecimen	12.7 (26)	72.2 (148)
5. I would give consent to use my identifiable health information or biospecimen for research only on my specific health/illness issues *		
health information	37.2 (77)	42.5 (88)
biospecimen	37.1 (76)	39.0 (80)
6. I need to know the identity of the person to whom my identifiable health information/biospecimen is being given before I would give consent *		
health information	54.1 (112)	22.7 (47)
biospecimen	59.8 (122)	19.1 (39)
7. I am comfortable with medical researchers using my de-identified health information or biospecimens without my consent		
health information	23.9 (49)	65.4 (134)
biospecimen	21.4 (44)	62.6 (129)
8. I believe I should be given the opportunity to change my mind and take back my consent once I have given it *		
health information	15.5 (32)	62.3 (129)
biospecimen	13.7 (28)	63.2 (129)
9. I would like to know when my identifiable health information or biospecimens are used for medical research		
health information	22.7 (47)	50.7 (105)
biospecimen	35.3 (72)	44.1 (90)
10. I would like to know when my de-identified health information or biospecimens are used for medical research		
health information	39.3 (81)	35.9 (74)
biospecimen	46.6 (96)	33.0 (68)

*P* < 0.005

*significant at *p* <0.005 (Chi Square)

### Qualitative data

Thirty-three people provided brief comments. The main theme arising was that of altruism and desire to contribute to better care for others by permitting the use of their information or specimens for research. *(“I feel if my specimens are of benefit to finding cures or improving quality of life for our community then it should be freely used;” “If others could benefit from the use of my information, it would be a good thing. I have no problem with it”)*.

People also commented on the value and importance of research (“*I feel research is necessary to advance the medical field…”*), the preference that consent be sought (*“….the individual still has the right to choose”*) and a few comments reflecting concern that uses of information might not be congruent with individual beliefs or may be used by others such as insurers or governments *(“The nature of the research is important to me; it does not have to be about my illness or condition however, there are types of research I would not support;" “I believe medical research and use of personal health info and biospecimens is necessary and useful. However it needs to be handled responsibly. I would not want the info to be used in other areas to separate or disqualify in other areas such as insurance policies").*

For a few people, while consent was not believed to be necessary, being advised their information had been used was important *(“I would like to know if my information is being used. It would make no difference why or to what research its being applied”).*

## Discussion

In general, these patient participants were supportive of medical research and very trusting of medical researchers. Most believed that consent should be sought for use of health information or biospecimens and most indicated they would always give consent for any medical research. This is consistent with the reports of others [11, 17, 21]. While some participants have discussed the need to retain control over their information or material through a consent process, our findings suggest that for a substantial proportion of people, knowing their information or biospecimens were being used for research purposes was sufficient. As noted by others, a wide range of opinions was given, making it difficult to suggest a single approach to engaging people in sharing of these valuable research resources [16, 18, 21].

The overall response rate for the study was 24.9 %. This increased to 52.8 % for those who agree to take the survey package for consideration. Correspondingly, the findings are potentially limited by the respondents who were already positively disposed towards research self-selecting into the study. Together with these respondents being from a unique patient population in a single clinic, the results may not be representative of the general population.

### Health information

Congruent with the provisions of the Health Information Act, over two thirds of the sample appreciated that consent requirements could preclude, or limit the usefulness of, medical research. The remaining participants either disagreed or did not hold an opinion. Despite the background information provided, this may reflect limited understanding of research and concomitant privacy standards, suggesting a need for public education in this area. Limited patient or public understanding of research and the use of health information has been previously reported with similar calls for education arising [13, 15, 19].

Contrasting with provisions of the Health Information Act, two thirds of the sample believed in the absence of their own ability to give consent for health information access, it should be permissible for consent to be obtained from a family member (i.e., a surrogate). Currently, unless there is a specific, written authorization in place to this effect (i.e., a research directive) the HIA prohibits consent from another s104(i), leaving it to REBs to consider granting a waiver where people lack capacity or are otherwise unable to consent. Whereas surrogate decisions might be made based on what they believe their family members would have wanted or possibly their family member’s best interests, REBs make the decision based on the feasibility of obtaining an individual consent. If the prospective participant cannot make this decision due to limitations of capacity, or due to mortality, it is clearly not feasible to obtain their consent. In such circumstances, REBs must favour the utility of non-consensual data use, where public interest outweighs individual privacy interests, if the information is to be released. While this is intended to maximize the good coming from use of this information, it does so at the expense of individual preference as understood by a surrogate. Although this survey sought opinion on the validity of surrogate consents, it did not assess people’s awareness of the current standard. A better understanding of pubic perceptions would inform future public education and legislative revisions.

More than half of those expressing an opinion about the form consent should take agreed that it could be oral as opposed to written. This preference is again at odds with the provisions of the Health Information Act, although as with surrogate consent, it is consistent with the TCPS2. Alignment of these regulatory requirements would facilitate more consistency in the application and acceptance of research ethics standards.

While the Health Information Act requires a specific research purpose be given for the use of health information (s34), over half of respondents indicated they did not need to know the exact purpose or had no opinion. Even fewer people seemed interested in this level of detail for biospecimen use. This suggests that most people are comfortable providing a broad, or general consent and is consistent with the reports of others [15, 18]. In line with our findings, the US Department of Health and Human Services (HHS) advanced changes to the Health Insurance Portability and Accountability Act (HIPAA) in 2013 that allow “compound authorizations” (i.e., consents) for the use of health information for future, unspecified research purposes, as long as a reasonably informed decision can be made [26]. In contrast to use being for any purpose whatsoever (i.e., a blanket consent), providing a general research direction is viewed as sufficient to permit a valid exercise of autonomy (i.e., a broad consent).

An inconsistency was observed in opinions on the need for consent and use of deidentified data. While most respondents indicated that “individual” consent should always be sought for the use of de-identified health information (i.e., Table 3, item 6), most also indicated they personally (i.e., “I”) were comfortable with use of their de-identified information without their consent (i.e., Table 4, item 7). This may reflect respondents making a distinction between the “other” and the self on this issue, or it may reflect an ambiguous opinion. This finding has implications for the wording of future survey items and is an area worth exploring further.

### Biospecimens

Consistent with the above and with current guidance under TCPS2 (2014), most participants were comfortable with the nonconsensual use of deidentified biospecimens. However, over one-third of those responding were either unsure or were not comfortable with this lack of consent (Table 4, item 7). Taken together with a significant proportion of people indicating they would like to know when their deidentified health information or biospecimens are used for research, such discomfort raises the possibility of public backlash at the presumptive use of their information, should they become aware of such activities. Without transparency in the research process, either through the provision of information that deidentified data or biospecimens are being used or a formal consent process, there is the potential for individuals to feel that research is being undertaken in secrecy. This perception could foster mistrust, ultimately undermining public support of future research. The United States Department of Health and Human Services released a Notice of Proposed Rule Making (Sept 8, 2015) that, if brought into effect, would mandate consent for use of de-identified biospecimens [7]. However, as with health information, broad consent would be acceptable, permitting future unspecified uses on the basis of the initial consent. Permitting broad consents seeks to balance individuals’ rights to authorize use of their personal health data/biospecimens while reducing the burdens for both researchers and participants associated with obtaining project-specific (i.e., narrow) consent.

### Health information and biospecimens

The results on research use of biospecimens compared to health information revealed statistically significant differences in opinions. These differences were not consistently more restrictive for one source of data vs. the other. It is not clear how relevant or meaningful these differences are in reality, given the general similarity in proportions. Addressing this directly in future research would be informative.

## Conclusion

This study described the opinions of patient participants on the research use of their health information and biospecimens considered in the context of current standards.

Clinical health data and biospecimens are valuable research resources, critical to the advancement of medicine. Use of these data for research requires balancing respect for individual autonomy, privacy and the societal interest in the greater good. Both the TCPS2 and the Health Information Act recognize this trade-off by providing processes where the usual consent requirements can be waivered or altered. This research revealed that these participant members of the public did not always share the positions on consent that are upheld by the HIA and the TCPS2.

Research using clinical data, including health records and biospecimens has increased dramatically over the past decade and greater consideration is being given to the rights of those who provide these research resources. Recognizing that respecting the individual preferences is not always feasible, these findings have implications for the regulation of research access to health information and biospecimens. If regulations are intended to reflect the preferences of the majority of population they are intended to protect, Canadian standards should be reviewed, with consideration given to permitting broad consents and to requiring consent for access to de-identified biospecimens. Finally, public education should be encouraged to a greater extent so that understanding, transparency and trust is fostered regarding research uses of health information and biospecimens.

## Abbreviations

HHS, Health and Human Services; HIA, Health Information Act; HIPAA, Health Insurance Portability and Accountability Act; TCPS2, Tricouncil Policy Statement 2

## Additional files

Additional file 1: Patient perspectives on the research use of health information and biospecimens. Survey tool. (DOCX 29 kb) Additional file 2: Background information. Information provided to participants to assist with survey completion. (DOCX 97 kb)
