# Supplementary material for: A survey of patient perspectives on the research use of health information and biospecimens
Source: BMC Med Ethics. 2016 Aug 15;17:48. doi: 10.1186/s12910-016-0130-4 (PMC4986353; doi:10.1186/s12910-016-0130-4)
Supplement: Additional file 1: — Patient perspectives on the research use of health information and biospecimens. Survey tool. (DOCX 29 kb) [file 12910_2016_130_MOESM2_ESM.docx]

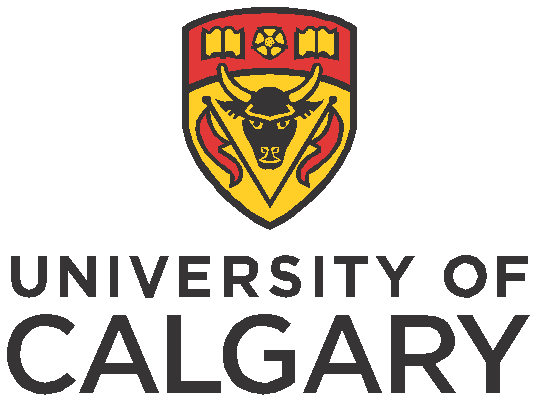


**Background Information**

Personal health information includes details about the diagnoses, treatments and care you have received, as well as details such as your name, address and date of birth. Health care professionals, like doctors and nurses, need this information to provide care to you.

Biological specimens, or ‘biospecimens’ like blood, urine, and other tissue samples (e.g., skin, organ, tumor) are sometimes taken from patients for clinical, diagnostic purposes (e.g., lab tests). Leftover samples are stored and can be used for research. Both biospecimens and personal health information can be de-identified so that research can be done without revealing information about individuals. To do this, personal details like names, addresses and dates of birth, are removed from the rest of the health information.

People have the right to keep all their information confidential. However, medical advances depend on the research use of personal health information and biospecimens. This allows researchers to gain understanding about the causes of diseases or to develop better treatments. In many cases, researchers will ask people

for their consent to use their information and biospecimens for research. However, in certain circumstances, researchers may want to use these things without the individual’s permission.

Research Ethics Boards must review all research proposals before researchers are allowed access to personal health information and biospecimens. A Research Ethics Board includes people who are familiar with research, law and ethics (e.g., doctors, nurses, lawyers, researchers, ethicists, community members). The Board reviews researchers’ requests for access to identifiable information and biospecimens and decides whether or not the research should be allowed to go ahead, and whether or not the patient’s consent must first be obtained. The Board must balance respect for individuals’ right to give their own consent with the possible benefits to society that may arise from allowing access to personal health information and biospecimens without consent. In making its decision, the Board considers the importance of the research question, the qualifications of the researcher,

the safeguards in place to protect the information and whether or not it is reasonable or feasible for researchers to obtain consent.

Sometimes, researchers would be unable to do the research if they were required to get consent from every person whose health information or biospecimens they wanted to use. For example, there might be a very large number of people needed for the research making it too costly or too time-consuming to find everyone and ask for consent. In other situations, people might have moved away or, for other reasons, might be unavailable to give their consent. In these cases, important research might not get done if individual consent was required.

Although Research Ethics Boards have some policies and guidelines to help them with decisions about release of personal health information and biospecimens without consent, it is important to know what people in the community think about this.
