# Supplementary material for: A survey of patient perspectives on the research use of health information and biospecimens
Source: BMC Med Ethics. 2016 Aug 15;17:48. doi: 10.1186/s12910-016-0130-4 (PMC4986353; doi:10.1186/s12910-016-0130-4)
Supplement: Additional file 2: — Background information. Information provided to participants to assist with survey completion. (DOCX 97 kb) [file 12910_2016_130_MOESM1_ESM.docx]

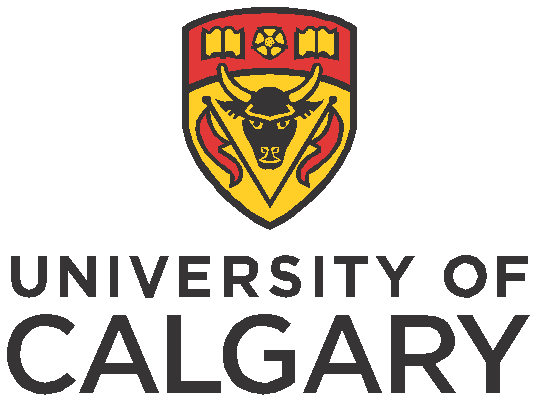


PATIENT PERSPECTIVES

on the research use of health information and biospecimens

**Please read the background information sheet and complete the questions in the pages that follow. If you finish the survey while in the clinic, please put it back in the yellow envelope and return to**

**clinic sta! or to the study box. If you need to take it home, please mail the finished survey back in the white, postage-paid envelope provided.**

The following questions relate to your perceptions, opinions and experiences of research, use of personal health information, biospecimens and consent. Please answer each question to the best of your ability. There are no right or wrong answers and you do not have to answer questions that you do not want to.

If you would like to remain anonymous, please do not put any personal/identifying information (like your name) on this survey.

**1. Have you ever taken part in medical research other than this survey?**

O yes

O no (please go to question 3)

O I’m not sure (please go to question 3)

**2. If yes, what did the research involve? (please check all that apply)**

O a drug or other medical treatment (e.g., counseling, exercise program)

O imaging information (e.g., x-rays, MRIs, CT scan)

O use of my tissue samples (e.g., blood, urine, stool, tumor)

O completing surveys, questionnaires or interviews

O use of my personal health information (e.g., chart or medical records)

O I don’t remember

O other: please specify

**3. General opinions on research, consent and use of health information**

The following questions ask for your opinions about research, consent and use of health information for medical research. In all cases, please assume that your health information would be protected and kept secure with the strongest data and privacy protections. When data are de-identified, it means that the researchers would not know from whom the information came.

**St ly d**

| **rongly**  **isagree** | **Disagree** | **Neutral** | **Agree** | **Strong**  **agree** |
| --- | --- | --- | --- | --- |
| O  O | O  O | O  O | O  O | O  O |
| O | O | O | O | O |

Sometimes, medical research cannot be undertaken without using individual’s

identifiable health information.

Promoting good medical research is an

important goal for the community.

I trust Research Ethics Boards to make decisions

about when individual’s consent for use of

their identifiable health information for research is necessary.

**Strongly disagree**

**Disagree Neutral Agree**

**Strongly agree**

Sometimes, getting an individual’s consent to

use identifiable health information for research might be so difficult that it would prevent the research

| O  O | O  O | O  O | O  O | O  O |
| --- | --- | --- | --- | --- |
| O | O | O | O | O |
| O | O | O | O | O |
| O | O | O | O | O |
| O  O | O  O | O  O | O  O | O  O |
| O | O | O | O | O |
| O | O | O | O | O |
| O | O | O | O | O |

from being done.

There are risks from researchers using identifiable

health information.

There are benefits from researchers using identifiable

health information.

I trust medical researchers to use identifiable health

information respectfully.

If consent can only be obtained from some individuals and therefore only some information can be gathered, the research results might not be

as useful.

Individuals should always be asked if their

de-identified health information can be used for medical research.

Individuals should always be asked if their identifiable health information can be used

for medical research.

Consent must be obtained in writing or by electronic

means (i.e., by email) for it to be OK.

It is OK to obtain consent verbally (i.e., over the

phone, or in face-to-face discussion)

Where individuals are unable to provide their own consent, it is OK to get consent from a member family member (e.g., spouse/partner, offspring)

**4. Opinions on consent, use of personally identifiable and de-identified health information.**

The following questions ask about your preferences for consent and use of your health information for medical research. As above, in all cases, please assume that your health information would be protected and kept in accordance with the highest data security and privacy protections possible. When data are described as de-identified, this means that the researchers would not know who the information is about.

**Strongly**

**disagree**

**Disagree Neutral Agree**

**Strongly**

**agree**

I would allow medical researchers to use my identifiable health information for any medical

research purpose without my consent.

O O O O O

I would always give medical researchers consent to use my identifiable health information for any

medical research purpose.

O O O O O

O O O O O

I am comfortable with medical researchers using my

de-identified health information without my consent.

I need to know the exact medical research purpose for which my identifiable health information is being

used before I would give consent.

O O O O O

It is enough for me to know my identifiable health information is being used for medical

research in general.

O O O O O

I would consent to have my identifiable health information held in a research repository, or “data

library” and used as needed by researchers.

O O O O O

I would give consent to use my identifiable health information for research only on my specific

health/illness issues.

O O O O O

I need to know the identity of the person to whom my identifiable health information is being given

before I would give consent.

O O O O O

I believe I should be given the opportunity to change my mind and take back my consent once I

havegiven it.

O O O O O

O O O O O

I would like to know when my identifiable health

information is used for medical research.

I would like to know when my de-identified health information is used for medical research.

O O O O O

**5. Opinions on the use of biological specimens (biospecimens)**

In addition to personal health information, researchers sometimes use extra, or left-over, biospecimens that are not needed for lab tests or other clinical reasons.

The following questions ask about your preferences for consent and use of your biospecimens for medical research. As above, in all cases, please assume that your biospecimens would be protected and kept in accordance with the highest data security and privacy protections. When biospecimens are de-identified, it means that the researchers would not know from whom they came.

**Strongly**

| **disagree**  O | **Disagree**  O | **Neutral**  O | **Agree**  O | **agree**  O |
| --- | --- | --- | --- | --- |
| O | O | O | O | O |
| O | O | O | O | O |
| O | O | O | O | O |
| O | O | O | O | O |
| O | O | O | O | O |
| O | O | O | O | O |
| O | O | O | O | O |

**Strongly**

I am equally concerned about the research use of my identifiable biological specimens and the research

use of my identifiable health information.

I am more concerned with the use of my identifiable biological specimens for research than I am with the

use of my identifiable health information.

I would always give medical researchers consent to use my identified biological specimens for any

medical research purpose.

I am comfortable with medical researchers using my de-identifiable biological specimens without

my consent.

I need to know the exact research purpose for which my identifiable biological specimens are

being used before I would give consent.

It is enough for me to know my identifiable biological specimens are being used for medical

research in general.

I would consent to have my identifiable biological specimens held in a biobank and used as needed

by researchers.

I would give consent to the use of my identifiable biological specimens only for medical research related to my specific health/illness issues.

**Strongly**

| **disagree** | **Disagree** | **Neutral** | **Agree** | **agree** |
| --- | --- | --- | --- | --- |
| O | O | O | O | O |
| O | O | O | O | O |
| O  O | O  O | O  O | O  O | O  O |

**Strongly**

I need to know the identity of the person to whom my identifiable biological specimens are being given

before I would give consent.

I believe I should be given the opportunity to change my mind and take back my consent once

I have given it.

I would like to know when my identifiable

biospecimens are used for medical research.

I would like to know when my de-identified

biospecimens are used for medical research.

**6. Please use the space below to provide any comments you may have on medical research and use of personal health information and biospecimens.**

**7. Please provide the following demographic information. You do not have to answers any questions that you do not want to.**

7.1 Gender:

O Male

O Female

7.5 Ethnic background

7.2 Year of birth: 19

7.3 Highest level of education completed

O less than high school O high school diploma O some post-secondary

O technical School/ College diploma complete

O European Canadian

O Asian Canadian O African Canadian O First Nations

O other (please describe: )

O prefer not to answer

7.6 Have you ever had a kidney biopsy?

O post secondary degree(s) complete

O prefer not to answer

O Yes

O No

7.7 How long have you been a patient at

this clinic?

7.4 Income Range:

O <$20 000

O $20 000- <$40 000

O $40 000- <$60 000

O $60 000- <$80 000

O $80 000- <$100 000

O >$100 000

O prefer not to answer

O this is my first visit O less than one year O 1-5 years

O 6-10 years

O more than 10 years

O prefer not to answer


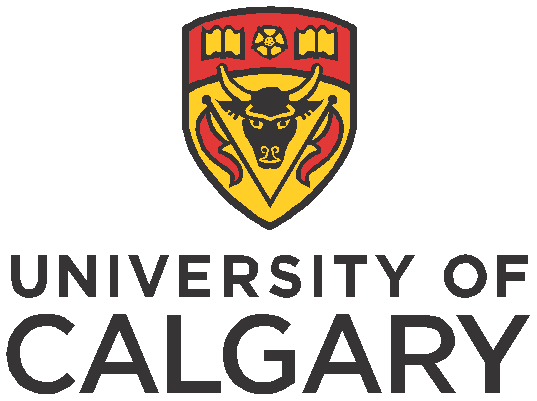
**Thank you for taking the time to complete and submit this survey.**
